# One-stage complete resection of giant intracardiac leiomyomatosis with moderate hypothermia extracorporeal circulation and beating heart technique with 36 months follow-up—a case report

**DOI:** 10.1186/s13019-016-0445-8

**Published:** 2016-04-12

**Authors:** Xihui Li, Feng Xiao, Yinmo Yang, Yindong He, Siyu Zhang

**Affiliations:** Department of Cardiac Surgery, Peking University First Hospital, Beijing, China; Department of General Surgery, Peking University First Hospital, Beijing, China; Department of Gynaecology, Peking University First Hospital, Beijing, China

**Keywords:** One-stage resection, ICL, Beating heart, Follow-up

## Abstract

**Background:**

Intracardiac leiomyomatosis (ICL) is a rare benign neoplasm of the smooth muscle in the uterus extending into the heart. Complete resection is difficult because of the extensive range.

**Case Presentation:**

We report a case of one-stage complete resection of a giant ICL with moderate hypothermia extracorporeal circulation and beating heart technique.

**Conclusions:**

The outcome of 36 months follow-up was very good.

## Background

Intravenous leiomyomatosis (IVL) is a rare benign neoplasm of the smooth muscle in the uterus. It can extend into the inferior vena cava (IVC), right atrium, right ventricular and pulmonary artery. It is called intracardiac leiomyomatosis (ICL) once the heart is involved. A radical operation is difficult because of the extensive range. We report a case with ICL in which one-stage radical resection under moderate hypothermia extracorporeal circulation and beating heart technique was performed.

## Case presentation

**Fig. 1:**
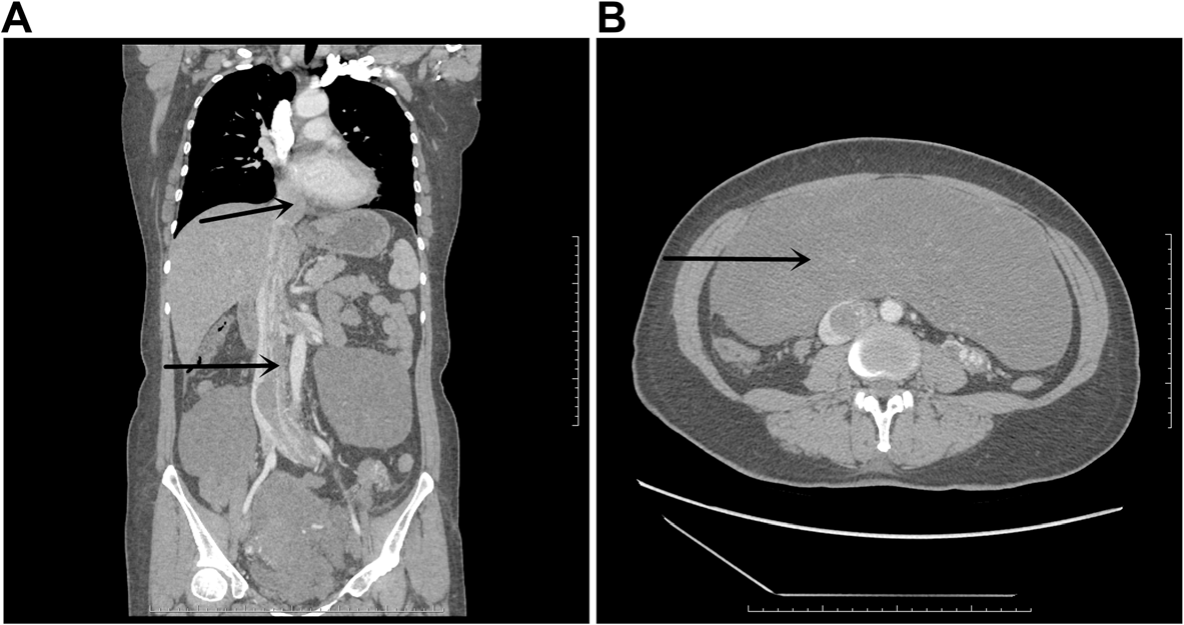
Enhanced computerized tomography (CT) shows the tumor encroached on the IVC, right atrium and right ventricle (**a** *arrows*). The tumor in the pelvic cavity is huge (**b** *arrows*)

A 44-year-old woman was admitted with 3-year history of edema of the double lower extremities and one-day history of a large abdominal mass. Enhanced computerized tomography (CT) of the abdomen and thorax revealed a uterine mass of 23.9 cm*8.7 cm*23.8 cm (RL*AP*SI) in size and encroached on the double vena ovarian veins, double common iliac veins, IVC, right atrium and right ventricle (Fig. 1). Echocardiography showed a free-floating low-level echo mass in the right atrium, right ventricle and the main pulmonary artery, moderate tricuspid regurgitation, and moderate pulmonary hypertension. Based on these findings, a diagnosis of ICL was made. The patient was scheduled for a one-stage operation under extracorporeal circulation.

**Fig. 2:**
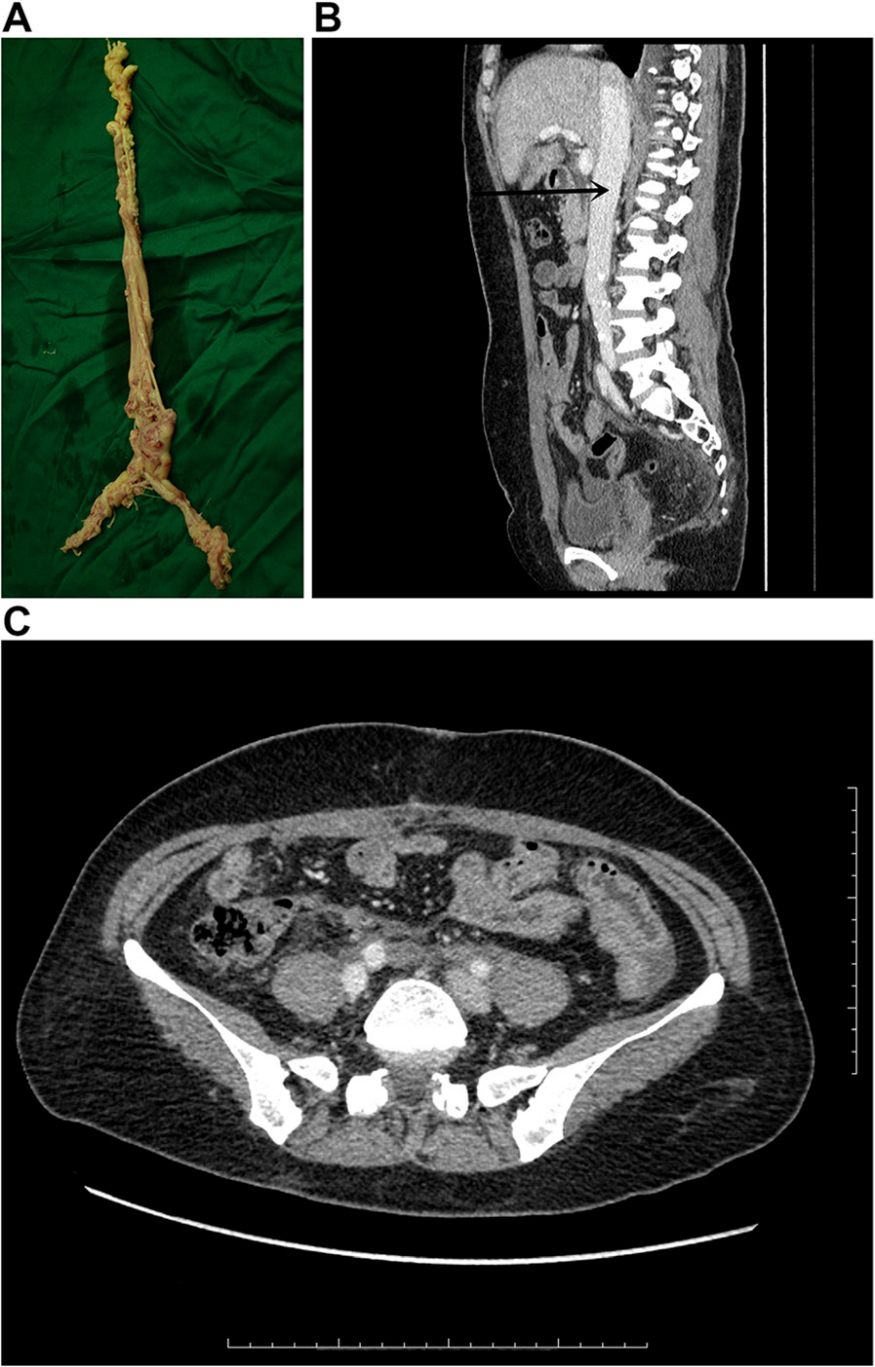
The tumor in the double common iliac veins, IVC, and heart was resected (**a**). The CT after operation showed complete resection of the tumor (**b, c**)

A laparotomy was performed by two gynecologists and a general surgeon. Total abdominal hysterectomy with bilateral salpingo-oophorectomy including the tumor mass was performed. Then a median sternotomy was made. Cardiopulmonary bypass (CPB) was established between the ascending aorta and the right atrium. A straight venous cannula was inserted into the right atrium carefully and the top of the cannula was pointed to the superior vena cava (SVC) to avoid tumor falling off. After initiating the CPB, the nasopharyngeal temperature was decreased to 27–28 °C and the flow rate was decreased too. Ventricular fibrillation occurred when the temperature reached 27–28 °C. Then, the venous cannula in the atrium was inserted deeper into the SVC, and the right atrium was opened safely. The intracardiac mass was free-floating without adhering to the cardiac structure and was easily extracted from the right ventricle. The inferior intracaval mass was attached to the wall of the IVC. The attachment sites were dissected, and the intracardiac and intracaval portions were completely resected. The residual tumor in the double common iliac veins was entirely removed. The incision of IVC and common iliac veins were continuously sutured. The patient was rewarmed and ventricular fibrillation stopped as the temperature was increased. Then the CPB was disconnected slowly, and the total duration of CPB was 130 min. Massive transfusion was needed during the operation. The pathology result confirmed the diagnosis of IVL with extension into the heart (ICL). The patient was discharged on the 15th day post-operation without any complications. The CT after operation showed complete resection of the tumor (Fig. 2). Anti-estrogen therapy was not given due to complete resection. One year warfarin therapy was used to maintain international normalized ratio (INR) of 1.5 to 2.0 because of the huge wound of IVC. No residual tumor recurred at 12-month, 24-month and 36-month follow-up. The patient has a normal life.

## Conclusions

IVL is a rare benign tumor that originates from the uterine venous wall or uterine leiomyoma. Sometimes it can extend into the heart and is called ICL. It is potentially underdiagnosed. Prompt diagnosis combined with surgical excision can increase the survival rate and result in good prognosis [1]. A case of a 40-year-old asymptomatic woman with incidental finding of a cardiac murmur was previously reported. Urgent cardiac surgery revealed an ICL, extending from the IVC and involving all four cardiac chambers and the aortic root [2]. The radical resection of ICL is very difficult and complicated because of the extensive range. One-stage and two-stage operations have been reported [3–6]. A retrospective study of 20 patients with ICL reported that nine patients underwent one-stage operations and 11 patients underwent two-stage operations. There was no significant difference in the postoperative complications between the two groups [7]. Complete resection was recommended for both single and two-stage operations in a review of 182 patients. Once complete resection is achieved, recurrence is rare [8]. Incomplete removal leads to recurrence in one-third of the patients [9].

In our patient, one-stage operation was considered though the uterine mass was huge because it has several advantages: 1. Quick elimination of the symptoms; 2. Avoidance of pulmonary embolism between the two stages; 3. One-time usage of general anesthetic; 4. Avoidance of excessive growth of the mass; 5. Alleviation of psychological burden of waiting for the second operation; and 6. Reduction of financial burden. The patient’s general status was very good, and there was extensive cooperation between the cardiac surgeons and gynecologists in our hospital. And our patient was quickly discharged without any post-operative complication.

A review of 182 patients showed one-stage operation in 37.9 % patients, two-stage operation in 36.8 % and other approaches (including cardiac only, declined surgery, not documented) in 25.2 % patients [8]. One-stage operations have been increasingly reported from 2000 to 2015. Management of CPB during the operations includes CPB with cardiac arrest [10–15] or without cardiac arrest (with beating heart) [3, 16, 17].

During our operation, the temperature was decreased to moderate hypothermia and ventricular fibrillation occurred. Echocardiography showed a free-floating mass in the heart before the surgery so we predicted a minor intracardiac operation and used the beating heart technique. Ventricular fibrillation occurred but stopped automatically when the body was rewarmed. This technique simplified the procedure. The advantage of moderate hypothermia was that the flow of CPB could be reduced to half. Once the IVC was opened, the hemorrhage could be reduced and the manipulation was easy.

The positive outcome proved that hypothermia CPB with beating heart technique was optimal. With this technique, we could achieve complete resection and simplify the procedure.

### Consent

Written informed consent was obtained from the patient for publication of this Case report and any accompanying images. A copy of the written consent is available for review by the Editor-in-Chief of this journal.
